# Immunometabolic Links between Estrogen, Adipose Tissue and Female Reproductive Metabolism

**DOI:** 10.3390/biology8010008

**Published:** 2019-02-07

**Authors:** Sally A. Eaton, Jaswinder K. Sethi

**Affiliations:** 1 Human Development and Health, Faculty of Medicine, University of Southampton, Southampton SO16 6YD, UK; s.eaton@soton.ac.uk; 2 National Institute for Health Research Biomedical Research Centre Southampton, University Hospital Southampton NHS foundation Trust, Southampton General Hospital, Southampton SO16 6YD, UK; 3 Institute for Life Sciences, University of Southampton, Highfield, Southampton SO17 1BJ, UK

**Keywords:** estrogen, sex-dependent adiposity, metabolic flexibility, reproductive metabolism, metabolic syndrome, metaflammation, immunometabolism, menopause, obesity, metabolic syndrome

## Abstract

The current knowledge of sex-dependent differences in adipose tissue biology remains in its infancy and is motivated in part by the desire to understand why menopause is linked to an increased risk of metabolic disease. However, the development and characterization of targeted genetically-modified rodent models are shedding new light on the physiological actions of sex hormones in healthy reproductive metabolism. In this review we consider the need for differentially regulating metabolic flexibility, energy balance, and immunity in a sex-dependent manner. We discuss the recent advances in our understanding of physiological roles of systemic estrogen in regulating sex-dependent adipose tissue distribution, form and function; and in sex-dependent healthy immune function. We also review the decline in protective properties of estrogen signaling in pathophysiological settings such as obesity-related metaflammation and metabolic disease. It is clear that the many physiological actions of estrogen on energy balance, immunity, and immunometabolism together with its dynamic regulation in females make it an excellent candidate for regulating metabolic flexibility in the context of reproductive metabolism.

## 1. Introduction

Many of the physiological differences that exist between men and women are linked to their differing reproductive roles. The differences manifest beyond the reproductive tissues and are intricately involved in both whole-body energy metabolism and functionality of a healthy immune system. In viviparous females, the former ensures appropriate provision of energy, nutrients, and building blocks for a potential pregnancy, the developing fetus, labor and during lactation; while the later prevents immune rejection of the semi-allogeneic fetus. Reproductive success is further guaranteed (or prevented), by complex signaling networks involving multiple organs, that integrate and link environmental, nutritional, and hormonal cues to fertility. An overarching feature is the appropriate coordination of reproductive metabolism. Given its central role in energy metabolism, it is now clear that adipose tissue is a major player in regulating sexually dimorphic physiology, displaying the greatest number of sexually differentiated transcripts of all human tissues after mammary glands and skeletal muscle [1]. It is also a major site in which the metabolic and immune systems interact and in pre-menopausal women, may hold important clues for the natural mechanisms that protect against obesity-linked metabolic disease. This review aims to focus on adipose tissue and how the systemic immune-metabolic actions of the sex hormone estrogen, mediates its role in supporting reproductive metabolism. However, other metabolically relevant organs also exhibit sexual dimorphism and are likely involved in integrated regulation of reproductive metabolism. Excellent reviews have recently been published on sex-dependent central regulation of appetite and energy expenditure [2]; liver metabolism [3], and muscle physiology [4] and complement the current review.

From a mechanistic perspective, sexual dimorphism in adiposity has been linked to two principal determinants: Sex chromosomes and sex hormones, and modern technological advances are allowing us to probe in greater detail the molecular players and signaling networks involved. One elegant model which highlights the influence of sex chromosomes is the FCG (four core genotypes) mouse model in which the gonad-determining gene, *Sry* has been moved to an autosome and thus mice can be generated to have one of four combinations of sex chromosome and gonads. Subsequent characterization of this model demonstrated a two-fold higher total fat mass in mice with two X chromosomes, independent of gonadal steroids [5]. Additionally, the number of X chromosomes may itself influence adiposity rather than the absence of Y chromosome [5]. In contrast to the limited studies on sex chromosomes, our understanding of the role of sex hormones (particularly estrogen) in adiposity is relatively more advanced. This is principally driven by an interest in improving our understanding of why menopause is associated with increased adiposity and greater risk of developing metabolic disease. However, at puberty, a surge of sex hormones creates sexually diverse phenotypes and while this remains generally constant for males, females experience notable fluctuations in their sex hormones throughout life in response to estrous cycles, during pregnancy, labor, and lactation, which then cease at menopause. These normal fluctuations are themselves linked to significant metabolic changes as described in rodent studies [6]. Moreover, since menopause is linked to redistribution of fat stores, promoting larger visceral adipose depots, it is likely that sex hormones, such as estrogen, play a critical role in regulating metabolic flexibility to support changing demands in reproductive females. In this review we discuss the recent advances in our understanding of physiological roles of estrogen in regulating (a) healthy adipose tissue distribution, form and function; (b) normal immune function and, (c) the potential protective nature of estrogen signaling in pathophysiological settings such as obesity-related metabolic disease. 

## 2. Estrogen and Sex-Dependent Adipose Tissue Distribution, Form, and Function

Estrogen acts both centrally and systemically to regulate energy balance. Centrally it acts to regulate food intake and central mechanisms of energy expenditure. Systemically, it has been linked to a number of physiological features of adipose tissue form and function. These include: Limiting total adiposity, regulating adipose tissue distribution, and adipocyte function (i.e., promoting lipolysis and thermogenesis).

### 2.1. Estrogen and Fat Storage

Both the total amount of adiposity and differential distribution of fat in specific depots are well characterized and sex-dependent differences outside of gonadal differences have been discussed in detail elsewhere [7,8,9]. In brief, although adult women have a greater percentage total fat mass, men tend to accumulate greater amounts of visceral adipose tissue, found around the gonads and abdomen, whereas pre-menopausal women tend to store more fat in subcutaneous depots such as the gluteo-femoral and mammary depots [10]. After menopause, or in conditions where estrogen is low, a greater proportion of fat is stored in central fat depots [11,12,13]. Pre-menopausal women are also resistant to complications arising from diet-induced obesity, including insulin resistance and metaflammation [14]. This is generally also true for other mammals including laboratory model organisms i.e., mice and rats. From the perspective of reproductive metabolism, the need for greater amounts of energy storage (in white adipose tissue) is a clear prerequisite to meet the energetic demands of pregnancy, labor, and lactation [15]. However, the need for differential tissue distribution is less clear. One possibility could be the need to store surplus energy in locations that are unlikely to impede on space requirements for a growing fetus. It is also likely that fluctuating metabolic requirements associated with reproductive cycle, pregnancy, labor, and lactation are linked to fuel storage in depots that are most responsive to fuel mobilizing cues i.e., are more metabolically flexible. This is consistent with the observation that estrogen depletion (e.g., post-menopause) results in both fat redistribution and metabolic inflexibility. 

The amount of total body fat is itself associated with reduced metabolic flexibility, at least in healthy men where it has been investigated [16]. Metabolic flexibility can be defined as the ability of an organism to adapt its fuel selection following changes in its availability or nutritional status i.e., from a fed to a fasted state [17]. It is also highly relevant to switching of energy demands that accompany pregnancy, labor, and lactation. Although body weight is negatively correlated with metabolic flexibility, it has been found that women, who have a greater percentage fat mass, have greater metabolic flexibility than men, and that the amount of visceral adipose tissue (VAT) in both men and women negatively correlated to metabolic flexibility [10]. Moreover, estrogen administration to aged male mice improves metabolic flexibility [18]. However, further studies are needed on the role of estrogen and metabolic flexibility in females, particularly in post-menopausal models and subjects.

Mechanistic elucidation of the role of estrogens in regulating adiposity has come about through numerous studies examining models and conditions which alter the amount of sex hormones. During menopause the level of circulating estrogen dramatically drops, and this is thought to drive increased visceral adipose depots and increased risk of metabolic disease. This redistribution of fat storage is reproduced in ovariectomized (OVX) mouse models [7,19,20,21]. These models of circulating estrogen deficiency all show increased body weight, increased fat depot weight (particularly in visceral depots), and importantly, these features are ameliorated by exogenous administration of estrogen [19,21,22]. Moreover, further enhancing estrogenic activity, by transgenic over-expression of steroid sulfatase in adipose tissue of female mice, limits diet-induced obesity specifically in visceral but not subcutaneous depots. This phenotype is also reversed following ovariectomy [23]. Estrogen has also been found to regulate bone marrow adipocytes with increased fat mass observed in OVX and in ERα knockout (KO) mice compared to OVX + estradiol (E2) or wildtype mice respectively [24]. In humans, estrogen therapy also prevents increase in bone marrow fat mass in menopausal women [25,26].

The estrogenic effects on adiposity requires a functional estrogen receptor alpha (ERα/ESR1). In rodent models with whole body ablation of ERα, both female (and male) mice exhibit a greater than two-fold increase in adiposity, particularly in the visceral fat depots [27,28]. Similarly, intra-abdominal fat pad-specific knockdown of ERα (using siRNA) also results in heavier fat pads with enlarged adipocytes (independent of sex). Furthermore, adipocyte-specific ERα deletion results in an increased total fat mass and gonadal fat mass in females and poorer glucose handling in male mice. Both sexes show increased gonadal adipocyte size with females and males showing three and two times greater hypertrophy compared to wildtype littermates, respectively [28]. That similar effects of ERα depletion are observed in both sexes is explained in part by the fact that mature adipocytes also synthesize estrogen from androgens (including testosterone) through the activity of aromatase. Indeed, mice lacking aromatase (encoded by the *Cyp19* gene) also exhibit increased intra-abdominal adipose tissue mass [29]. However, adipose tissue (in male but not female) mice can also inactivate estrogen via the activity of estrogen sulfotransferase (EST/*Sulte1*) [30]. Moreover, the expression of EST is induced by testosterone [30].

Local adipocyte-derived estrogen may also play a role in preventing preadipocyte differentiation—a possible mechanism for context-dependent titrated adipose tissue expansion [31,32]. In vitro studies have demonstrated that estrogen treatment inhibits adipogenesis and lipogenic gene expression [33]. Estrogen also stimulates preadipocyte proliferation via MAPK-dependent and c-fos signaling pathways [34]. Observational studies have compared preadipocytes from non-obese men and women and found, approximately 10% more early stage preadipocytes in the stromal vascular fraction of abdominal subcutaneous adipose and about 35% more in the femoral depots in female adipose tissue [35].

### 2.2. Estrogen and Fuel Mobilization

Estrogenic signals are implicated in the regulation of fuel mobilization and energy expenditure (i.e., lipolysis and thermogenesis). Differences exist between men and women in basal lipolysis rates relative to resting energy expenditure, and differences in the rate of lipolysis has been observed to be depot-specific, with stimulated lipolysis being greater in women’s abdominal subcutaneous adipose tissue (SAT) but greater in men’s VAT [36]. In addition to suppressing lipogenesis and lipogenic gene expression (i.e., inhibiting adipogenesis), estrogen is reported to promote both basal and catecholamine-induced lipolysis [37], with a reduction in lipoprotein lipase activity observed in women with low estrogen levels [13]. However, this contrasts with a recent study using pair-fed OVX versus OVX + estrogen supplemented groups and surgical controls, which found no effect of estrogen on lipolysis in either visceral or subcutaneous depots by a number of different measures [38]. When considering the potential role of estrogen in metabolic flexibility of white adipose tissue, it is clear that additional context-dependent variables remain to be fully addressed.

Nonetheless, estrogenic signaling has also been implicated in promoting thermogenic adipose tissue, since pre-menopausal women have more brown adipose tissue (BAT) and more active BAT than men or post-menopausal women [39]. Estrogen has been reported to increase BAT lipolysis, thermogenesis, and thus energy expenditure in several different rodent models [40,41,42,43]. Estrogen acts both centrally and systemically to regulate adrenergic signals and BAT activation. The former has recently been reviewed in depth [44]. However, the few studies using murine models of estrogen deficiency have reported a number of conflicting findings with respect to BAT activation; no change in BAT was observed in the ERα KO mice. In contrast, the Aromatase KO mice, an increase in BAT mass was observed, which was reversed by estrogen administration [45], and a rat OVX model had reduced BAT, which was also reversed by estrogen administration [46,47]. 

Recent findings have also confirmed a higher browning capacity in female murine peri-ovarian white adipose tissue (WAT) compared to male epididymal WAT [48]. Estrogen can directly activate beiging of WAT—a process also linked to catecholamine activity and increased energy expenditure. In vitro treatment of preadipocytes with an estrogenic agonists increased *Ucp1* expression along with several other markers of beige adipocytes, whilst reduction of ERα by 40% using RNAi reduced the agonist-driven increase of Ucp1. In addition, in response to cold exposure ERα KO mice have significantly less BAT activity than wildtype mice, while reintroduction of ERα to one inguinal WAT depot of female ERα KO mice results in an increase in beige-like characteristics of this depot [49]. The recent demonstration that thermoneutral housing conditions can be used for sex-independent modelling of non-alcoholic fatty liver disease in rodents [50] further underscores the need for careful monitoring and reporting of experimental variables such as housing temperatures. Indeed, future in vivo studies may require a more detailed systems biology approach that considers central and systemic effects and experimental variables such as housing conditions. This may help to reconcile conflicting data currently cluttering the literature on estrogen and energy expenditure.

## 3. Estrogen and Sex-Dependent Immunity

Sex-dependent differences in innate and adaptive immunity have long been implicated in the susceptibility to infection and immune-related disorders, for example men are more likely to succumb to bacterial infection and sepsis, and mortality of malignant cancer is twice as high in men [51]. Conversely, women are up to 80% more prone to auto-immune diseases such as multiple sclerosis, systemic lupus erythematosus (SLE), and rheumatoid arthritis [51,52]. At a physiological level, and in the context of reproduction in viviparous mammals, host-defense mechanisms in females are thought to have adapted to prevent host rejection of developing fetus.

Previous studies have amassed a substantial amount of conflicting data relating to sex hormone-dependent inflammatory responses. However, recent studies are beginning to address potential reasons for the conflicting data. By performing detailed temporal profiling of the inflammatory response to specific stimuli, it appears that females exhibit a greater initial but much more transient pro-inflammatory response. This is followed by a robust anti-inflammatory response. In contrast, males present with a persistent pro-inflammatory response, which may leave them more vulnerable to sepsis and inflammatory diseases [51,53,54]. 

Mechanistically, sex-dependent regulation of the duration of an inflammatory response has been reported to be influenced by both sex chromosomes and sex hormones, including estrogen. Investigations using the FCG mice (described above) found either the X or Y chromosome to stimulate the immune system to greater effect depending on the disease model. The X chromosome conferred a greater immune response on SLE prone mice [55,56] but OVX mice exposed to an autoantigen presented with a greater immune response in mice carrying the Y chromosome, which was then reversed upon administration of testosterone [57]. The pattern recognition receptor TLR7 is located on the X chromosome and is thought to escape some X inactivation with higher expression of TLR7 in women’s peripheral blood leukocytes, which drives increased production of IFN-α thereby promoting a greater auto-immune response [58].

However, estrogen has also been reported to play a significant role in regulating innate immune response to infection and inflammation, although it has been shown to have both pro- and anti-inflammatory effects depending on a number of factors including but not limited to: Concentration of estrogen, target organ, type of immune cell target, type of immune stimulus, time since infection, and expression of receptors [59]. In general, in response to LPS (Lipopolysaccharides) challenge, male and OVX female mice have higher levels of pro-inflammatory cytokines such as IL-6 and IL-1β [60,61,62] than female mice, or OVX mice receiving estrogen [62]. In addition, estrogen pre-treatment of LPS-stimulated human monocytes (from male and female blood) inhibits the upregulation of the pro-inflammatory cytokine IL-6 [63]. Interestingly adipose-ERα knockout mice have increased mortality from bacterial uterine infections compared to wild-type mice [64]. Lastly, menopause or surgically-induced menopause has been shown to increase the amount of pro-inflammatory markers, which can be ameliorated by subsequent administration of estrogen [65,66], whilst treatment of estrogen to ovariectomized mice increased the expression of anti-inflammatory M2 macrophage markers thereby promoting resolution of inflammation [67]. However, as mentioned above not all studies have found estrogen to suppress pro-inflammation, Riant et al. found that estrogen supplementation in an OVX model increased pro-inflammatory markers [68] and high concentrations of estrogen in late pregnancy are associated with increased inflammatory TNFα levels [69].

In white adipose tissue, a nexus point for the immune and metabolic systems, estrogen has been found to suppress the pro-inflammatory response. Indeed, mouse models with either adipocyte- or macrophage-specific ERα ablation exhibit increased inflammation of their adipose tissue [28,70]. Conversely, increased estrogen bioavailability selectively in adipocytes (using the aP2_aromatase overexpression model) in male mice, results in reduced adipose tissue inflammation [71]. In vitro models also support this with treatment of estrogen to human monocyte-derived macrophages repressing the pro-inflammatory cytokines IL-6, IL-1β, and TNFα [72,73]. In addition, recent adipocyte and macrophage co-culture experiments have revealed that estrogen can help reduced the expression of pro-inflammatory cytokines challenged by conditions of metabolic stress (or after stimulation with TNFα). Estrogen, testosterone or DHT (dihydrotestosterone) treatments are all able to reduce the extent of inflammation [74,75]. Lastly, estrogen has also been shown to act on murine blood macrophages to shorten the pro-inflammatory response to stimuli, such as LPS, increasing the resolution of the inflammatory phase [76], which is consistent with estrogen action promoting a short but strong inflammatory response to infection which can then be followed by an anti-inflammatory response.

## 4. Estrogen and Sex-Dependent Differences in Obesity, Metaflammation, and Metabolic Disease

Obesity is associated with low-grade chronic inflammation, often termed metabolic inflammation or metaflammation, which is causally linked to obesity-associated metabolic diseases such as Type 2 diabetes, non-alcoholic fatty liver disease (NAFLD), cardiovascular disease, and certain cancers including breast cancer. Metaflammation, which is much more a feature in VAT in ‘unhealthy’ obesity, is characterized by the infiltration of adipose tissue macrophages (ATMs), in particular, pro-inflammatory macrophages, as well as other myeloid leukocytes. Collectively, these then produce greater amounts of pro-inflammatory cytokines, namely IL-1β, IL-6, and TNFα as well as chemokines such as MCP-1, which are causally associated with insulin resistance, dyslipidemia, and metabolic disease [77,78,79,80]. Studies in which obesity and inflammation have been segregated have shown that a reduction in metaflammation associates with improved insulin sensitivity [81,82,83].

In agreement with the higher susceptibility of both human and rodent males to metabolic disorders, as well as their larger VAT depots, males of both species have also been shown to have a greater inflammatory response to obesity [21,84,85]. In response to a high fat diet, male mice have significantly more pro-inflammatory ATMs, and increased expression of inflammatory cytokines than females [21,85]. Interestingly transfemales (males transitioning to females) who retained their testes and correspondingly have a much greater ratio of testosterone to estrogen had a greater incidence of insulin resistance than those who did not [86]. Further studies have revealed that male mice also have a greater expansion of myeloid precursors and that bone marrow cells transplanted from male mice into male or female recipients produced greater numbers of pro-inflammatory ATMs in response to a high fat diet compared to bone marrow cells from female donors. Intriguingly, this was independent of the sex of recipients, suggesting a role for hematopoietic cells in mediating the sexually dimorphic response to metaflammation [85,87]. 

Estrogen has also been heavily implicated in mediating the sexual dimorphism in metaflammation; OVX, ERα KO, and Aromatase KO mouse models have all shown that a reduction in circulating estrogen results in increased obesity-associated insulin resistance and metaflammation, with increases in pro-inflammatory ATMs and an upregulation of pro-inflammatory genes such as *TNFα*, *IL-6*, *IL-1β*, and *MCP-1* [19,21,28,88,89,90,91]. Studies on myeloid-specific ERα-KO mice have suggested that estrogen mediates significant metabolic effects, at least in part, through these cells. Mice lacking ERα exclusively in myeloid cells display increased adiposity, insulin resistance, and metaflammation [70]. Although much of the inflammatory cytokine production in adipose tissue is driven by macrophages, estrogen production, and estrogenic activity in adipocytes, can also regulate metaflammation in adipose tissue. Reduction of ERα specifically in adipocytes using adipocyte-specific AdipoCre knockout mice increases adipose tissue inflammation in both male and female mice. Male mice were more profoundly affected, possibly due to the sex-dependent differences in baseline levels of estrogenic activity [28]. In support of this finding, Ohlsson et al. found that increasing the amount of estrogen in adipose tissue of male mice resulted in a reduction of macrophage markers and immune cell infiltration, as well as an increase in insulin sensitivity [71]. However, it is likely that in addition to increasing estrogen in WAT and BAT, estrogenic activity was also increased in tissue resident macrophages of this model, further promoting the beneficial observations. Similarly, reduced crown-like structures and pro-inflammatory gene expression in adipose tissue, and improved insulin sensitivity are observed in obese mice with transgenic overexpression of estrogen activating enzyme, steroid sulfatase in the adipose tissue. These effects were reversed when the transgenic and wildtype mice were ovariectomized, identifying estrogen as the causative steroid responsible for preventing adipose tissue metaflammation [23].

## 5. Future Directions

Along with the three areas discussed above, there are several bourgeoning areas of research, which may provide some surprising insights into the mechanistic links between immunometabolism, adipose tissue, and reproductive metabolism. One such area is that of the gut microbiome, although it is now well established that the microbiome can affect adiposity [92,93]. Several studies suggest that the microbiome may also mediate sex-specific effects of adiposity and metaflammation. For example, one study investigated sex differences in the microbiome diversity and susceptibility to Type 1 diabetes in the NOD (Non-Obese Diabetic) mice. Under germ free conditions no sex-dependent differences were found in susceptibility to disease, but when housed in SPF conditions female mice had a greater susceptibility. If female mice received a microbial transfer from male mice, this gender difference in susceptibility was lost [94,95]. In another study OVX resulted in a shift in the ratio of the gut microbiome towards an obesity-like profile [96]. Another recent study has linked estrogen to mediating the ratio of obesogenic gut microbiome and consequently metabolic disease. Changes in gut microbiome and metabolic phenotype were observed in males and OVX females compared to females, OVX + E2 and males + E2. When female mice were given fecal transplants from males this difference in microbiome and metabolic phenotypes were then lost, while estrogen or estrogen-like compounds shifted the male metabolic phenotype and microbiome to that seen in female mice [97]. Further investigation is needed to establish how the gut microbiome is involved in regulating these sex-dependent differences (or vice versa) and if and how the adipose tissue-derived estrogen is involved in this interaction.

Another area to watch is that of extracellular vesicles (EVs), which are small membrane enclosed extracellular vesicles containing a mixture of selectively packaged RNA, DNA, and protein species and are capable of transporting their cargo from one cell (or organ) to another providing a vital form of intra- and inter-organ communication. EVs harvested from adipose tissue from obese *ob*/*ob* mice have been found to induce macrophage activation, proinflammatory cytokine release such as IL-6 and TNFα, as well as promoting insulin resistance when injected into wildtype mice [98]. Moreover, a recent study implicates adipocyte-derived microRNA-34a in mediating these paracrine actions on ATMs [99]. To date there have been a handful of studies which have identified differences in proteins associated with circulating EVs between men and women, as well as in different phases of the estrous cycle and between post-menopausal with or without estrogen replacement therapy [100,101,102]. One elegant study has investigated the miRNA content of serum exosomes in monozygous post-menopausal twins discordant for hormone replacement therapy and discovered six miRNAs that were significantly associated with serum 17β estradiol levels [103]. The authors also discovered adipose miRNAs discordant for these two groups [104] which, although not determined in the study, may be adipose-derived EVs. Further studies are warranted to determine how estrogen may regulate differences in EV content, if there are sex-dependent differences in adipose or macrophage EV content—particularly the regulatory RNAs, and if any altered content is involved in mediating the sex-dependent differences observed in progression of metabolic disease.

In summary, a complete elucidation of what drives the sex-dependent differences in metaflammation still has a long way to go. It is noteworthy that the current understanding has been limited in part, by the fact that most studies (a) do not report the sex of the model being investigated, or (b) are only conducted on male rodents or male cell lines, or (c) clinical studies often do not have sufficient power to distinguish sex-dependent differences in immunometabolic outcomes. Understanding how men and women respond differently throughout life and to differential energy demands will help to better inform more personalized healthcare. Indeed, there is already clinical evidence that women who receive estrogen replacement therapy are less likely to deposit adipose tissue in the intra-abdominal depot [105], and are relatively more protected from metabolic syndrome.
